# Conformational sampling and kinetics changes across a non-Arrhenius break point in the enzyme thermolysin

**DOI:** 10.1063/1.5130582

**Published:** 2020-02-14

**Authors:** Ming Dong, Mackenzie L. Lauro, Timothy J. Koblish, Brian J. Bahnson

**Affiliations:** 1 Department of Chemistry, North Carolina A&T State University, Greensboro, North Carolina 27411, USA; 2 Department of Chemistry & Biochemistry, University of Delaware, Newark, Delaware 19716, USA

## Abstract

Numerous studies have suggested a significant role that protein dynamics play in optimizing enzyme catalysis, and changes in conformational sampling offer a window to explore this role. Thermolysin from *Bacillus thermoproteolyticus rokko*, which is a heat-stable zinc metalloproteinase, serves here as a model system to study changes of protein function and conformational sampling across a temperature range of 16–36 °C. The temperature dependence of kinetics of thermolysin showed a biphasic transition at 26 °C that points to potential conformational and dynamic differences across this temperature. The non-Arrhenius behavior observed resembled results from previous studies of a thermophilic alcohol dehydrogenase enzyme, which also indicated a biphasic transition at ambient temperatures. To explore the non-Arrhenius behavior of thermolysin, room temperature crystallography was applied to characterize structural changes in a temperature range across the biphasic transition temperature. The alternate conformation of side chain fitting to electron density of a group of residues showed a higher variability in the temperature range from 26 to 29 °C, which indicated a change in conformational sampling that correlated with the non-Arrhenius break point.

## INTRODUCTION

Protein conformational changes are essential for enzyme function, and increased protein conformational flexibility has been linked to enhanced enzymatic activity.^1–8^ Protein flexibility has been described by conformational disorder, observed by crystallography or nuclear magnetic resonance (NMR),^8,9^ and by hydrogen/deuterium exchange (HDX) approaches.^10^ Notably, there have been studies utilizing HDX mass spectrometry that support a direct correlation between the time scale of conformational fluctuations and the turnover number of the enzyme thermolysin by showing that the substrate turnover is associated with the hinge bending that leads to a closed conformation.^11^

Furthermore, temperature has been used as a probe to study protein dynamics, and changes to structural stability and conformational dynamics of proteins have shown interesting results.^7,12,13^ Several enzyme systems have suggested that there is a biphasic transition in protein conformational sampling. Myoglobin showed extra mobility above −93 °C from neutron scattering results.^14^ Biphasic conformational dynamics behavior was observed from −53 to −23 °C in Zn-substituted cytochrome c peroxidase by studying the quenching of the ^3^ZnP excited state.^15^ In myoglobin and ribonuclease A enzyme systems, ligands were shown to bind to the protein only above a critical and specific temperature: −93 °C for myoglobin and −53 °C for ribonuclease A.^13,16^ In ribonuclease A, there was a biphasic break in the temperature dependence of the thermal B factor, indicating a biphasic protein dynamic behavior from −93 to −73 °C.^9^ Turning attention to a thermophilic enzyme, the alcohol dehydrogenase from *Bacillus Stearothermophilus* (ht-ADH), which is optimally active at 65 °C, also showed biphasic behavior in its Arrhenius plot between 30 and 40 °C.^17^ The origin of the non-Arrhenius behavior for ht-ADH was recently investigated by computer simulation and described as being based on a temperature dependence of the entropic contribution of the environment.^18^ The biphasic behavior observed in each system described above occurs in a temperature range below each enzyme's physiologically optimal temperature, and in each case, it has been implicated to mediate a functionally critical dynamic transition point of the enzyme. We predicted that thermolysin, which has optimal activity at 70 °C, would also exhibit biphasic non-Arrhenius behavior in the vicinity of room temperature. A previous temperature dependence of thermolysin kinetics, where the Arrhenius plot of *k_cat_*/K_M_ vs 1/T showed a non-linear relationship, was previously published, which supported this hypothesis, but was previously interpreted as a curved temperature dependence.^19^ In that paper, the authors assumed the K_M_ value of the substrate varies in a manner analogous to the Ki and measured the Ki vs 1/T, which was found to be linearly related. Therefore, the curve in the plot of *k_cat_*/K_M_ vs 1/T was mainly caused by the non-linear temperature dependence of *k_cat_*.

Room temperature crystallography has been applied to reveal motions crucial for catalysis, ligand binding, and the alternate conformation of protein side chains of interest.^9,13,20–22^ The program Ringer, which was developed for ambient temperature crystallography and alternate conformation interpretation, was used to discover hidden alternate conformations of side chains of a human proline isomerase CYPA from ambient temperature crystallography studies.^23^ The aim of the program Ringer is to go beyond static structural snapshots of proteins by uncovering structural ensembles in X-ray diffraction electron density maps. It functions by sampling electron density as a function of the side chain dihedral angles of a residue and identifies peaks that correlate with alternate conformations of the side chains. The program can detect hidden alternate conformations that could be significant for protein function. The hidden conformations identified using the program Ringer have provided clues to the functional roles of protein structural polymorphism and serves to assess the response of protein side chain distributions to perturbations including ligand binding, temperature changes, and mutations.^23^

In this study, we investigated a non-Arrhenius transition point in the kinetics of thermolysin at 26 °C that suggests a conformational sampling transition may exist at this temperature as well. To provide structural evidence to our hypothesis, we applied ambient temperature X-ray diffraction measurements to thermolysin across the temperature range of the enzyme's biphasic non-Arrhenius behavior using capillary mounted crystals and precise temperature-controlled data acquisition. An increased flexibility of the side chains of residues at the biphasic transition point indicated a change of the conformational sampling, which provides insight into how protein conformational sampling affects enzyme catalysis.

## MATERIALS AND METHODS

### Material for kinetic and structural analysis

Thermolysin from *Bacillus thermoproteolyticus rokko* used for kinetic analysis was purchased from Sigma-Aldrich, and the enzyme solution was exchanged into a reaction buffer containing 50 mM 2-(N-morpholino)ethanesulfonic acid (MES), pH 7.4, 1 mM CaCl_2_, and 4 M NaCl. Enzyme assays were performed with a thermolysin concentration of 0.16 *μ*M. The substrate furylacryloyl dipeptide N-(3-[2-furyl]acryloyl)-Ala-Phe amide (FAAFA) was purchased from Sigma-Aldrich, furylacryloyl dipeptide N-(3-[2-furyl]acryloyl)-Gly-Leu amide (FAGLA) was purchased from VWR, and a 10 mM stock solution of the substrate was prepared in Dimethyl sulfoxide (DMSO). A control was run using a range of DMSO concentration in the reaction solution, and the assay DMSO concentration was optimized to a value of 7% (*v/v*) to allow a better solubility of the substrate without compromising the enzyme activity. Thermolysin from *Bacillus thermoproteolyticus rokko* for crystallization was purchased from Hampton Research, and the protein stock solution was prepared by initially dissolving 50 mg of thermolysin into 1 ml of H_2_O, followed by two aliquots of 0.5 ml of 100 mM NaOH with gentle mixing to bring the solution to a total volume of 2.0 ml. Aliquots of protein were flash frozen in liquid N_2_ and stored at −80 °C.

### Kinetic assays

The furylacryloyl group of the FAAFA and FAGLA substrates permits the dipeptide hydrolysis rate to be readily monitored spectrophotometrically at 345 nm. The substrate FAAFA and FAGLA concentrations was measure using the ε_305_ = 25 000 M^−1^ cm^−1^. The activity was measured by the decrease in absorption Δε_345_ = −310 M^−1^ cm^−1^ by UV-VIS spectroscopy.^24^ The spectrophotometer was blanked with the assay buffer and two replicates were measured at each substrate concentration. The thermolysin kinetics assay with FAAFA as substrate was initiated by adding 0.2 *μ*M thermolysin to a solution of 50 mM MES, 1 mM CaCl_2_, 4 M NaCl,^25^ 7% (*v/v*) DMSO, at pH 7.5 after incubating the reaction sample in the water bath for 3 min. The kinetics assays were performed at a range of temperatures (19.0, 21.0, 23.0, 24.5, 26.0, 28.0, 30.0, 32.8, and 34.5 °C) controlled using a water bath ±0.1 °C. The FAGLA kinetics assay was performed at a range of temperatures (11.4, 13.2, 16, 17.7, 19.5, 22.8, 25.7, 29.2, 32.6, 38 °C) controlled using a water bath ±0.1 °C. At each temperature, we used a range of substrate concentration to measure initial velocity data, which was fit to the Michaelis-Menten equation. At each temperature assayed, the fitted value of V_max_ was used along with the concentration of thermolysin to calculate the rate constant *k_cat_*. The Arrhenius plot was drawn using the values of *k_cat_* at each temperature for the FAAFA substrate. For the FAGLA substrate, we used concentration of 0.5 mM which is at a concentration significantly lower than the estimated KM of 15–20 mM and the ln(*k_cat_*/K_M_) vs 1/T was plotted.

### Circular dichroism (CD) and fluorescence spectroscopy

Circular dichroism (CD) and fluorescence spectra were recorded for thermolysin over the temperature range of 20–33 °C. The protein samples were allowed to equilibrate at each temperature for 10 min before being analyzed. CD spectra were taken with a JASCO J810 spectropolarimeter with the protein solution contained in 0.1 cm path length cylindrical cell. Spectra were collected at 1.0 nm intervals over the wavelength range from 250 to 315 nm and a range of 195–250 nm. In each case, three scans were averaged for final spectra reported. Thermolysin was analyzed at 5 mg/mL under native conditions (10 mM phosphate buffer containing 50 mM NaCl, pH 7.5) for the wavelength range of 250–315 nm. A 10× dilution of the 5 mg/ml sample of thermolysin was prepared for the wavelength range of 195–250 nm. Mean residue molar ellipticity ([θ]) was calculated using [θ] = θ/nCl where n is the number of residues in thermolysin (n = 316), C is the molar concentration (dM), θ is the ellipticity in deg, and 0.l is the path length (cm). The ellipticity of the sample was corrected using the ellipticity of the buffer (10 mM phosphate buffer containing 50 mM NaCl, pH 7.5). The fluorescence spectra were recorded using an Aminco Bowman Series 2 fluorescence spectrometer for thermolysin samples at a concentration of 1 mg/mL in 50 mM MES buffer pH 7.4. The enzyme was excited at 280 nm and the fluorescence emission recorded at 333 nm was measured over the range of 23 °C–30 °C.

### Protein crystallization and capillary mounting of crystals

Protein crystals were grown using the hanging drop and sitting drop technique to obtain large and well diffracting thermolysin crystals using conditions obtained from protein crystallization screens from Hampton Research. Initial crystallization conditions of two crystallization conditions of thermolysin were as follows: (1) condition-1, Hampton Research Quick Screen Reagent C4, 1.4 M Na_2_KPO_4_ pH 6.9, and (2) condition-2, Hampton Research Crystal Screen HT Reagent H6, 1.5 M (NH_4_)_2_SO_4_, 12% glycerol, 100 mM Tris buffer, pH 8.5. Crystal-2 conditions were further refined to 1.4 M (NH_4_)_2_SO_4_, 12% glycerol, 100 mM Tris buffer, pH 8.3.

In order to grow larger crystals, the sitting drop technique was used by preparing each well with 600 *μ*l of crystallization solution, followed by adding 6 *μ*l of protein sample and 6 *μ*l of well solution sequentially to the sitting drop platform. After the solution was mixed, the wells were sealed and incubated at 25 °C. Once grown, thermolysin crystals were capillary mounted by pipetting crystals from the sitting drop platform into a 1.0 mm quartz capillary. After the crystallization solution was pipetted out using a 0.3 mm diameter capillary, both ends of the capillary containing the crystal were sealed with mineral oil and wax. In order to retain full hydration of thermolysin crystals during data collection, a small amount of residual crystallization solution was left inside the sealed portion of the capillary, yet not in direct contact with the crystal. Capillary mounted thermolysin crystals were stable and retained their diffraction properties following several weeks of room temperature storage.

### Ambient temperature data collection

Capillary mounted thermolysin crystals were used for ambient temperature X-ray diffraction data collection, positioned on the X-ray machine, and centered in the X-ray beam. The temperature was controlled with a modified water bath apparatus that kept the temperature of a compressed air stream to a set point (±0.1 °C) of data collections from 15 to 40 °C [Fig. 3(b)]. In the temperature control setup, the air nozzle was connected with copper coil which was submerged in the water bath apparatus. The key to its accuracy is due to a temperature probe at the outlet of the nozzle that provided feedback to the temperature-controlled water bath, thereby controlling the temperature of the X-ray diffraction data collection.

### X-ray diffraction data collection and structure determination

X-ray diffraction data sets were collected using a home source Rigaku RU-H3R/R-Axis IV instrument at a wavelength of 1.54 Å. The crystal to detector distance was 150 mm, and data sets were collected over 180 frames with 15 min 1° oscillations from one crystal at each temperature. The program HKL2000 was used to index, integrate, and scale the diffraction data.^26^

Structures of thermolysin in this study were solved by molecular replacement using the previously solved crystal structure of thermolysin (PDB code: 3DNZ) as the template. The program MOLREP of CCP4 was used for molecular replacement and REFMAC5 of CCP4 was used for refinements.^27^ Model building and modification were completed using the graphics program COOT.^28^ Water molecules were placed during successive cycles of model building and refinement. A 2F_o_–F_c_ electron density difference map for the model was used to confirm the validity of the entire model. The program PHENIX^29^ was used for the final crystal structure refinement of each data set.

### Protein data bank accession codes

The coordinates of thermolysin crystal structures reported have been deposited in the Protein Data Bank for data collection temperatures of 16.3, 19.5 21.0, 23.3 24.0, 26.3, 29.4, 35.2, and −180 °C with accession codes 5T9I, 5T9K, 5T9Q, 5TAC, 5TAD, 5TAE, 5TAI, 5TAJ, and 5TAK, respectively.

### Structural and conformational sampling data analysis

To characterize the alternate conformations of residue side chains of interest, we applied the program Ringer in our data analysis.^23^ The input files included the protein pdb file, the 2F_o_–F_c_ map file, the list file with the targeted residues information, and the configured Ringer input file. Parameters of the Ringer input files were configured by selecting the dihedral angle sampling degree and cutoff values. The output file from Ringer contained the electron density value for each increment around a targeted residue dihedral angle.

## RESULTS AND DISCUSSION

Protein dynamic and conformational changes as a function of temperature have been studied broadly by a variety of methods and have been detected in a variety of enzyme systems.^5,9,16,30,31^ In this study, we used temperature as a probe and studied thermolysin kinetic and structural differences to gain a fundamental understanding of what factors control the anomalous behavior in the enzyme's Arrhenius plot.

### Temperature dependence of kinetics of thermolysin

**FIG. 1.**
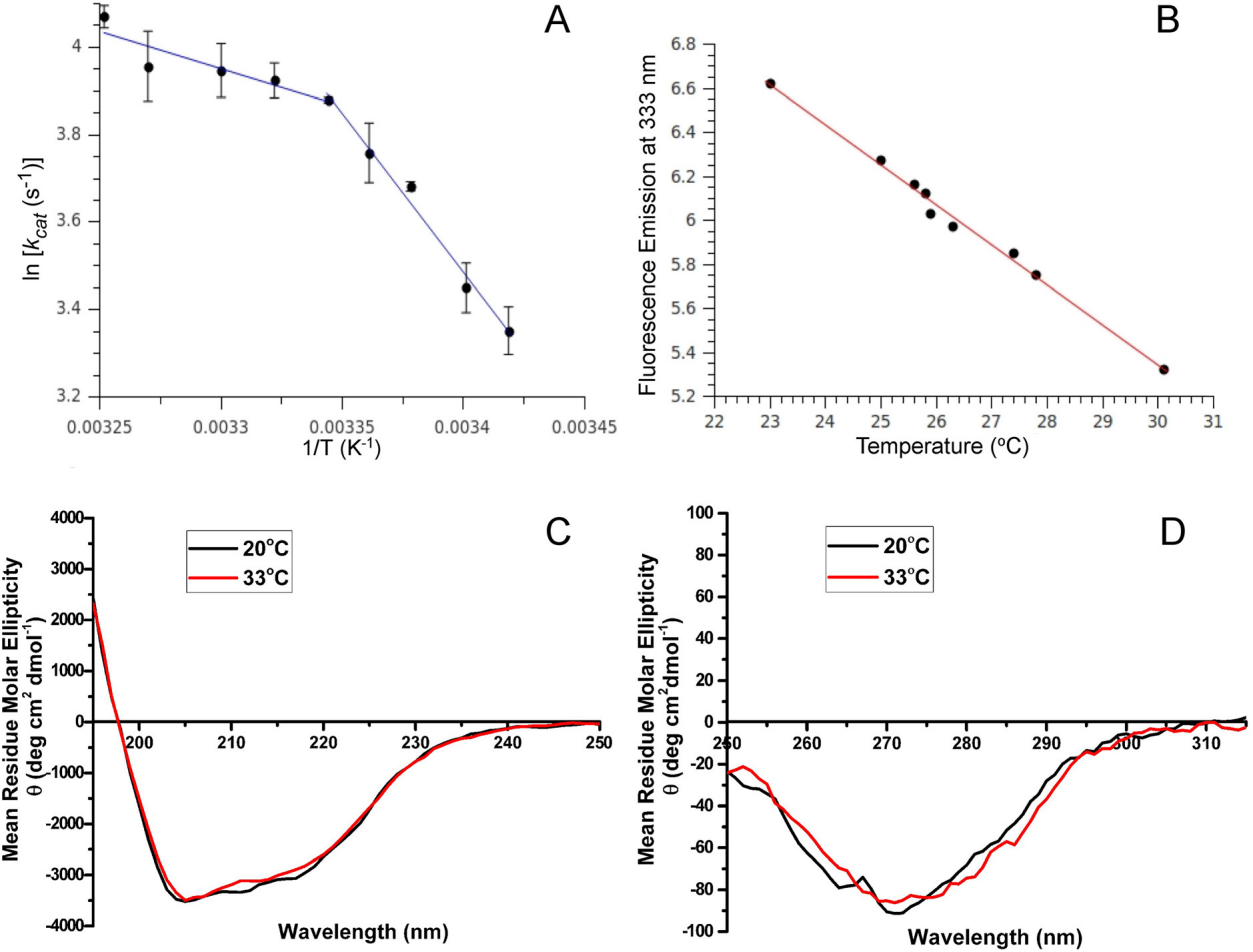
Kinetic and biophysical characterization of thermolysin across a temperature range that displayed a biphasic non-Arrhenius behavior. (a) Arrhenius plot of thermolysin hydrolyzing the substrate FAAFA. The plot of ln *k_cat_* vs reciprocal temperature for the thermolysin reaction showed a biphasic break at 26 °C. Both the activation energy E_a_ and A-factor have different values above and below this transition temperature. (b) Temperature dependence of fluorescence emission of thermolysin showed a linear relationship of the fluorescence emission at 333 nm and temperature, indicating there was no change to the oligomerization state of thermolysin in this temperature range. (c) A CD spectrum from 195 to 250 nm of thermolysin at temperatures above (red, 33 °C) and below the dynamic transition temperature (black, 20 °C) indicated no significant secondary structure change. (d) A near-UV CD spectrum from 250 to 315 nm showed no change of tertiary structure of thermolysin between 20 and 33 °C.

**FIG. 2.**
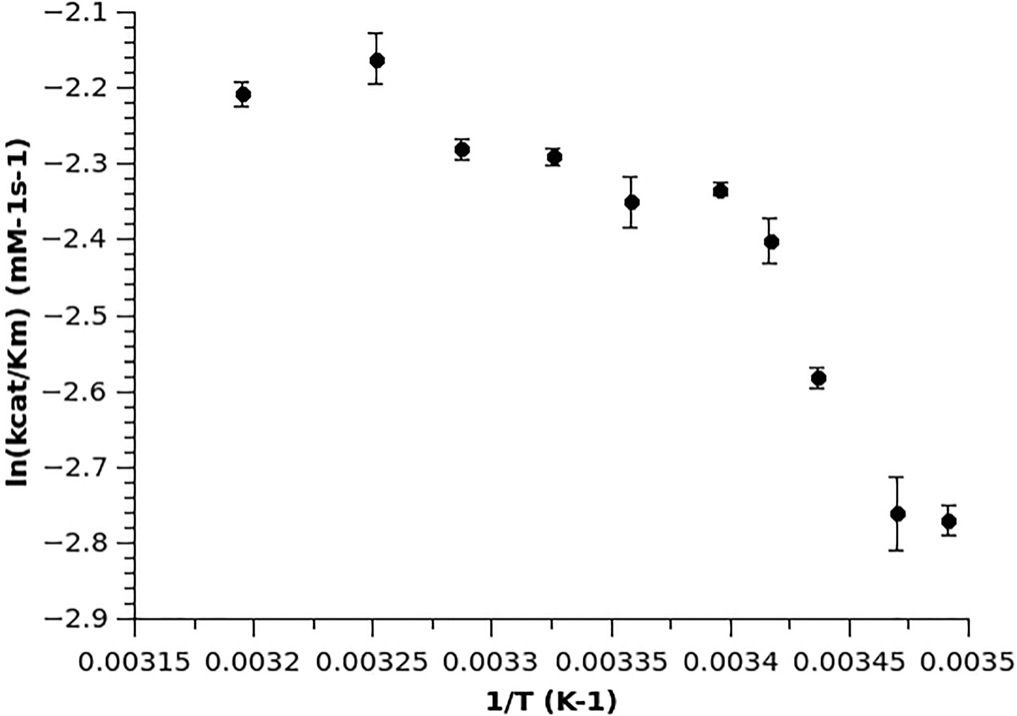
Kinetic characterization of thermolysin across a temperature range that displayed a non-linear Arrhenius behavior with another substrate. Arrhenius plot of thermolysin hydrolyzing the substrate FAGLA. The plot of ln *k_cat_* vs reciprocal temperature for the thermolysin reaction showed a biphasic break close to 26 °C. The activation energy E_a_ has different values above and below this transition temperature.

Previously Kunugi *et al.*^19^ reported a non-linear temperature dependence of *k_cat_*/K_M_ in the thermolysin system where the non-linear trend appeared at a temperature close to 30 °C. The trend had been fit with a spline curve, without a suggestion of non-Arrhenius behavior. Here we have extended this work, where we have shown a biphasic transition close to 26 °C from the Arrhenius plot of ln(*k_cat_*) vs 1/T for substrate FAAFA and ln(*kcat*/K_M_) vs 1/T for substrate FAGLA [Figs. 1(a) and 2]. The observed biphasic catalytic efficiency suggests a fundamental change of the protein above and below this transition temperature. From an analysis of the Arrhenius plot, both the activation energy (E_a_) and the Arrhenius-factor (A-factor) have different values above and below the transition temperature. The lower values of E_a_ and A factor above the transition temperature for FAAFA, and lower values of E_a_ above the transition temperature for FAGLA are consistent with a more optimized enzyme.

Compared to thermophilic ht-ADH, the value of the A-factor for thermolysin did not change as significantly^31,32^ when the temperature passed the transition point. However, results from thermolysin still indicate a functional and fundamental protein transition around 26 °C. The lower activation energy and A-factor above the transition temperature result in better catalytic efficiency and led to a hypothesis that the enzyme has different protein dynamics and conformational sampling above and below the transition temperature. More generally, our results with thermolysin further support a hypothesis that enzymes undergo a fundamental protein transition below their physiological and optimal temperature.

### CD and fluorescence spectroscopy of thermolysin

The steady state fluorescence emission of thermolysin at 333 nm demonstrated a red shift with increasing temperature [Fig. 1(b)]. Three Trp residues are positioned at different sites in thermolysin: one is close to the active site pocket, second is partially buried between two α-helices, and third is buried between an α-helix and a β-sheet. The linear relationship of the fluorescence emission and temperature observed suggested no biphasic structural or oligomeric changes of thermolysin across this temperature range. A CD scan from 195 to 250 nm collected at 20 and 33 °C, which spans the biphasic transition temperature of 26 °C [Fig. 1(c)], indicated that there was no detectable perturbation to the secondary structure of thermolysin. Additionally, a CD scan at 20 and 33 °C in the near-UV region of 250–315 nm indicated no detectable changes to tertiary structure occurred for thermolysin over this temperature range [Fig. 1(d)]. The fundamental change of the protein, which gives rise to the non-Arrhenius behavior and occurs at 26 °C, does not change the secondary or tertiary structure of the protein.

### Temperature dependent diffraction from two crystallization conditions

**FIG. 3.**
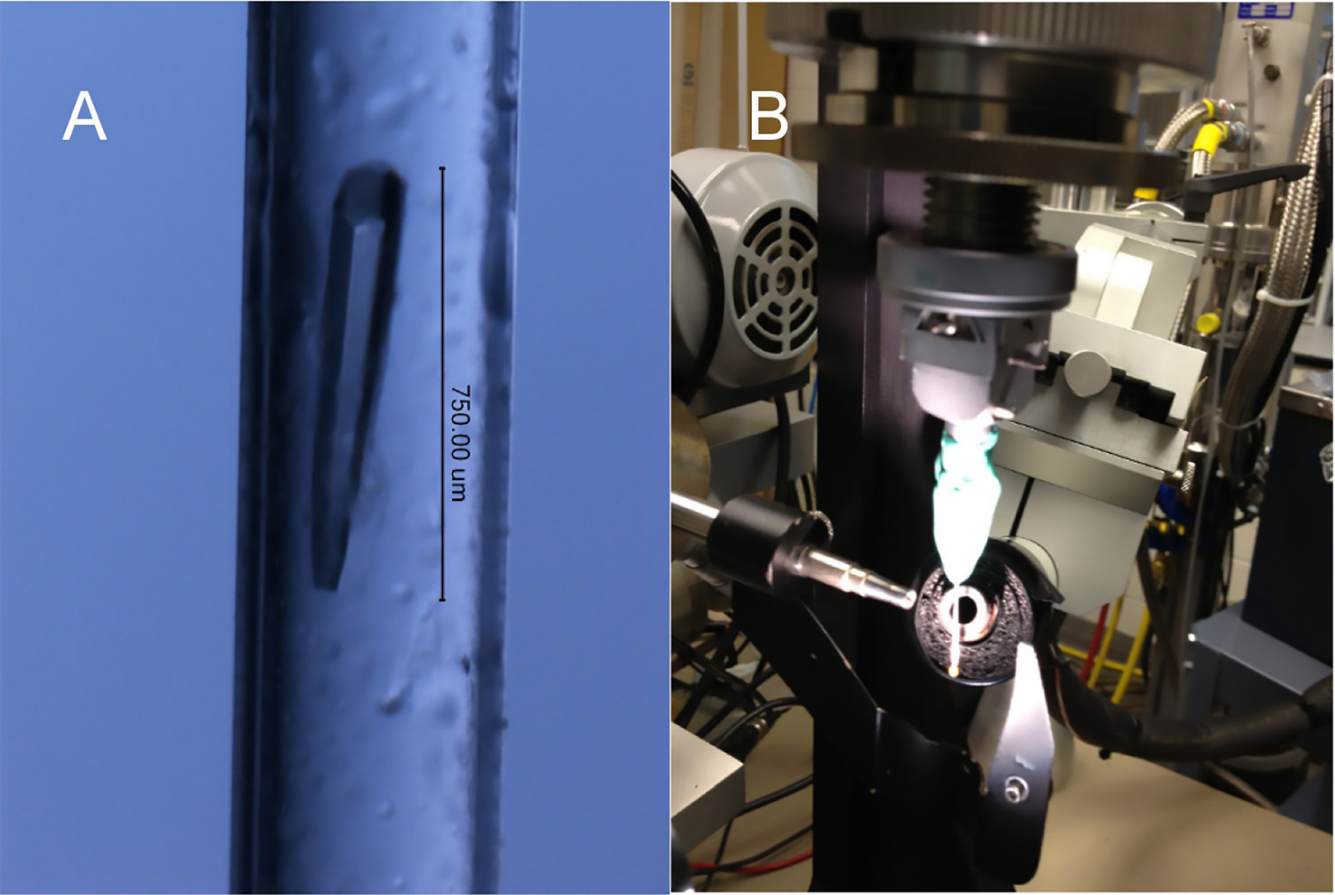
Ambient temperature X-ray crystallography data collection. (a) The thermolysin crystal was mounted in a quartz capillary sealed with mineral oil and wax, and small droplets of the crystallization solution were kept in the capillary to keep the crystal hydrated. Crystals used in data collections were typically 750 *μ*m in length. (b) Our ambient temperature X-ray data collection required the design and application of compressed air through a copper coil immersed in a temperature-controlled water bath, together with a feedback probe in the nozzle shown in figure, which allowed us to collect diffraction data sets over the range of 15–40 °C with an accuracy of ±0.1 °C.

After crystal quality evaluation and crystallization screening optimization at ambient temperatures using both sitting drop and hanging drop techniques two conditions were chosen to grow thermolysin crystals to be used for our experiments: crystallization condition-1: 1.4 M Na_2_KPO_4_ pH 6.9 and crystallization condition-2: 1.4 M (NH_4_)_2_SO_4_, 12% glycerol, 100 mM Tris buffer, pH 8.3 [Fig. 3(a)]. Both crystals grew in the hexagonal P6_1_22 space group, with identical unit cells and limits of resolution. However, the thermolysin crystals growing from these two conditions exhibited an altered tolerance to X-ray diffraction as a function of temperature. Crystallization condition-2 crystals showed robust X-ray diffraction across the full experimental temperature range and were used for data collection and structure solutions from 16 to 36 °C. In contrast, thermolysin crystals from crystallization condition-1 often lost high resolution X-ray diffraction at temperatures higher than the observed non-Arrhenius break point temperature of 26 °C.

### Thermolysin crystal structures at ambient temperatures

**FIG. 4.**
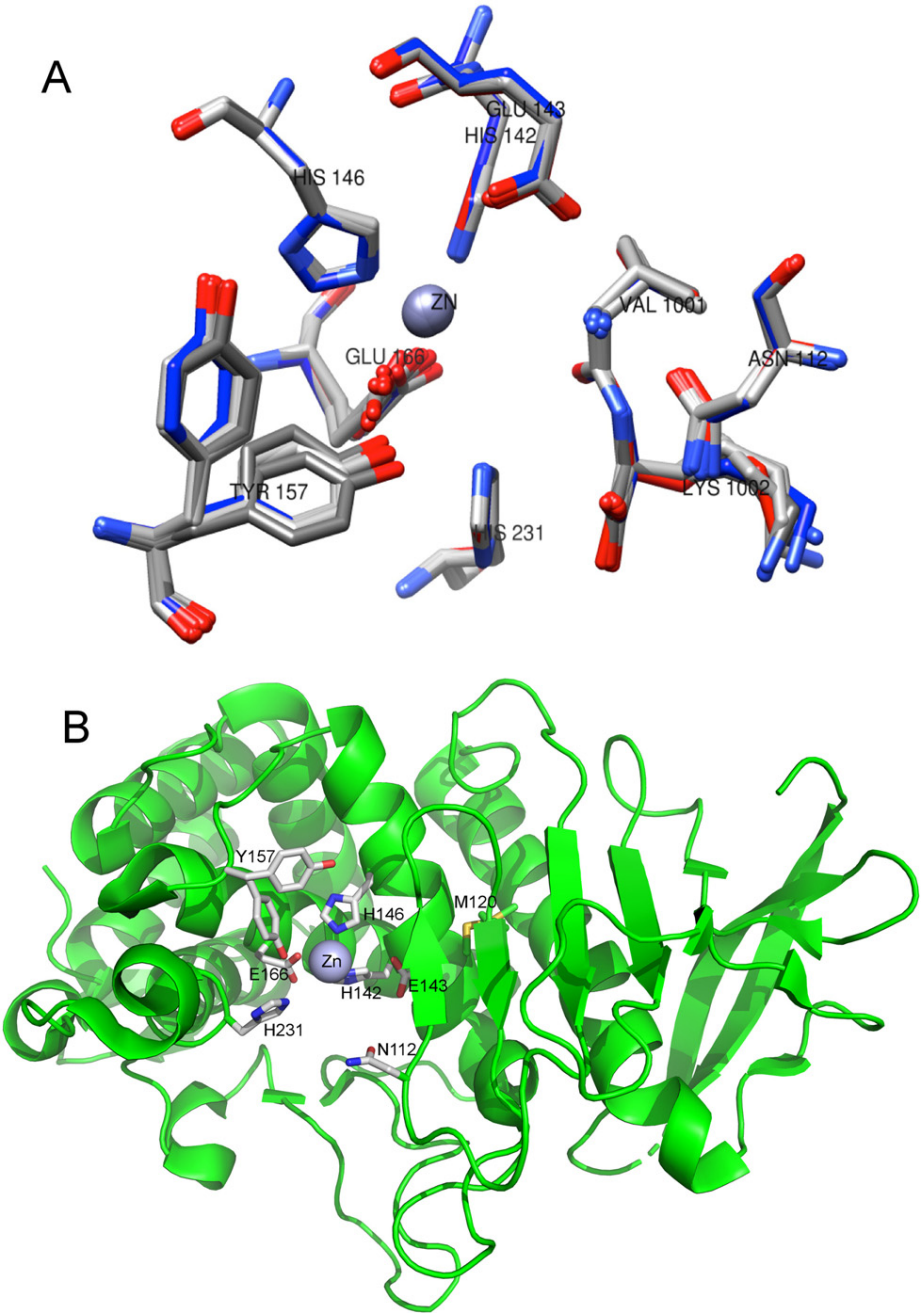
The structure of thermolysin showed small changes across the temperature range that displayed biphasic non-Arrhenius behavior. (a) Overlay of the active site side chains (collected at 16.3, 19.5, 21.0, 23.3, 24.0, 26.3, 29.4, 35.2 and −180 °C). Residue Y157 was modeled with two alternate conformations. The Zn binding residue E166 had a shifted *X_2_* angle in the temperature range studied, resulting in a carboxylate oxygen-Zn distance change from 1.93 to 2.20 Å. Overall, the side chains of the active sites shifted only slightly across this temperature range. The color scheme of residues is displayed by heteroatom. The bound product dipeptide Val-Lys is also displayed for these nine structures. (b) The active site side chains shown in panel A, along with M120 (displayed with two alternate conformations) of thermolysin are shown relative to its overall fold.

Capillary mounted thermolysin crystals diffracted up to 2 Å at ambient temperature. Thermolysin crystals grown from conditions of crystallization condition-1 and -2 were each indexed in the P6_1_22 space group and with cell dimensions a, b, and c of 93.7, 93.7, and 131.2 Å, respectively. The current model of each refined crystal structure contains 316 amino acid residues, four calcium ions, one zinc ion, and a bound product dipeptide Val-Lys for each subunit. A summary of the X-ray data collection and refinement statistics of eight crystal structures collected at 16–36 °C and one at −180 °C of crystallization condition-2 of thermolysin is listed in Table I. The refined structures showed very subtle differences as shown in the overlay of active site residues from the nine structures shown in Fig. 4.

**TABLE I.** X-ray diffraction data collection and refinement for thermolysin crystallization condition-2.

Space group	*P6_1_22*	*P6_1_22*	*P6_1_22*	*P6_1_22*	*P6_1_22*
Unit cell dimensions	93.7, 93.7,131.2	93.7, 93.7,131.2	93.7, 93.7,130.9	93.7, 93.7,131.2	93.8, 93.8,131.1
*a, b, c* (Å), α, β, ɣ (°)	90.0, 90.0, 120.0	90.0, 90.0, 120.0	90.0, 90.0, 120.0	90.0, 90.0, 120.0	90.0, 90.0, 120.0
Temperature (°C)	16.3	19.5	21	23.3	24
Resolution (Å)	50.0–2.0	50.0–2.1	50.0–2.1	50.0–2.0	50.0–2.09
Completeness (%)	99.6	100	97.4	100	97.5
Redundancy	17.7	18.1	7.7	16	15
*I/σI*	17.9	18.6	11.4	20.3	17
Resolution (Å)	44.1–2.09	40.58–2.1	40.58–2.1	31.97–2.04	44.15–2.09
*R_work_/R_free_*	0.145/0.196	0.138/0.195	0.15/0.206	0.136/0.186	0.147/0.197
Mean B value	28.1	27.5	24.9	26.2	27.6
Root mean square deviation (RMSD) bond lengths (Å)	0.019	0.021	0.021	0.018	0.021
RMSD bond angles (°)	1.37	1.25	1.25	1.27	1.26
Protein data bank (PDB) code	5T9I	5T9K	5T9Q	5TAC	5TAD
Space group	*P6_1_22*	*P6_1_22*	*P6_1_22*	*P6_1_22*	
Unit cell dimensions *a, b, c* (Å), α, β, ɣ (°)	93.8, 93.8,131.0 90.0, 90.0, 120.0	93.5, 93.5,130.7 90.0, 90.0, 120.0	93.3, 93.3,130.6 90.0, 90.0, 120.0	93.1, 93.1,129.7 90.0, 90.0, 120.0	
Temperature (°C)	26.3	29.4	35.2	−180	
Resolution (Å)	50.0–2.3	50.0–2.3	50.0–2.03	50.0–2.00	
Completeness (%)	97.9	100	99.8	99	
Redundancy	9.4	18.5	18.8	17.6	
*I/σI*	10.25	17.5	24.22	22.3	
Resolution (Å)	34.52–2.3	46.77–2.3	46.77–2.03	38.51–2.00	
*R_work_/R_free_*	0.151/0.22	0.145/0.23	0.145/0.202	0.188/0.261	
Mean B value	30.3	35.9	31.0	27.8	
RMSD bond lengths (Å)	0.019	0.02	0.02	0.02	
RMSD bond angles (°)	1.32	1.37	1.29	1.38	
PDB code	5TAE	5TAI	5TAJ	5TAK	

**FIG. 5.**
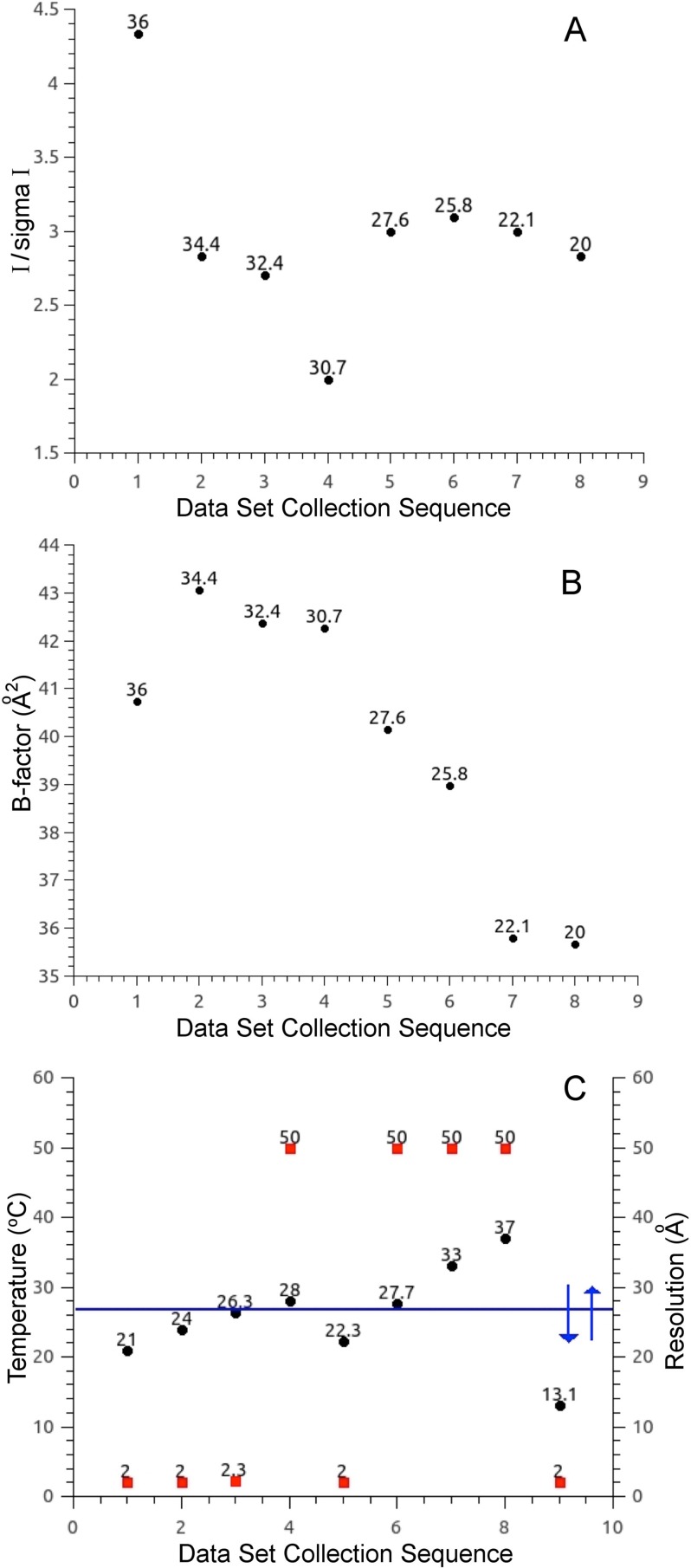
Multiple data sets from a single crystal revealed biphasic behavior. (a) Eight diffraction data sets of thermolysin from crystallization condition-2 were collected at the temperature shown in °C above each circle on the plot. The reflection intensity divided by its sigma is plotted on the y-axis for each successive data set. (b) The B-factor of each data set collected for the same single crystal shown in panel-A is plotted on the y-axis and the temperature is shown in °C above each circle on the plot. (c) A thermolysin crystal from crystallization condition-1 [different from crystallization condition-2 shown in panels-(a) and -(b)] diffracted well at or below 26.3 °C, but lost most of its diffraction (diffraction limit of 50 Å) when the temperature was at or above 27.7 °C. Interestingly, this process was shown to be reversible as the same crystal which had ceased to diffract above this temperature regained 2 Å diffraction when the temperature was brought below the biphasic non-Arrhenius break point (red solid squared represent resolutions of the crystals).

**FIG. 6.**
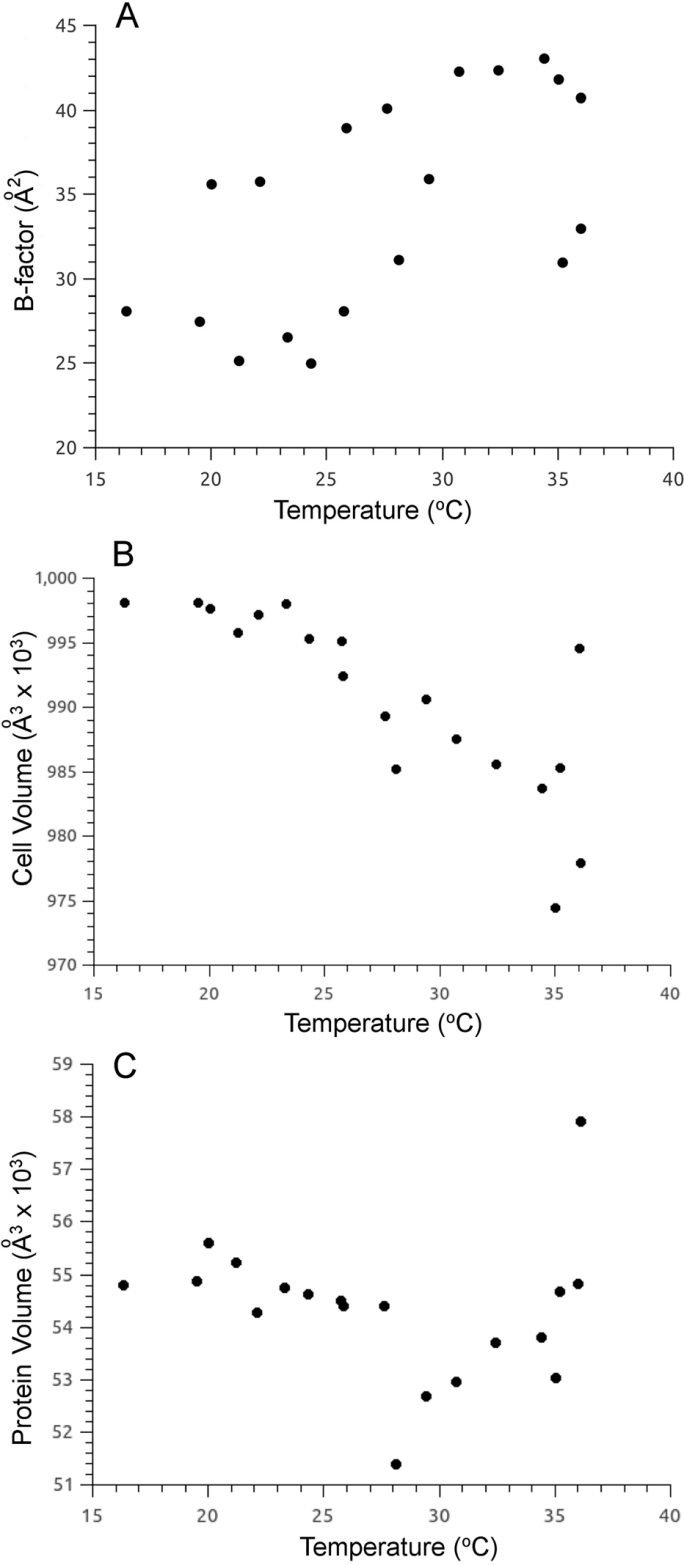
X-ray diffraction data that spanned the biphasic non-Arrhenius break point of thermolysin. Crystals grown from crystallization condition-2, which retained high quality diffraction below and above the biphasic non-Arrhenius break point, were used to collect 19 full data sets, which were used to assess changes across the temperature range of 16–36 °C. (a) The B-factor increased across the range of temperatures with no overall biphasic behavior confirmed. (b) The unit cell volume of the thermolysin crystal showed a linear decrease across the temperature range studied. (c) The protein volume of each thermolysin subunit did not change below and up to 27 °C, then followed an upward trend above this temperature.

Furthermore, the thermolysin crystals were robust to long term X-ray exposure. For example, a single crystal was used to collect eight consecutive data sets at varied temperature, showing that this crystal did not display significant deterioration of data quality [Figs. 5(a) and 5(b)]. X-ray diffraction data from multiple crystals of crystallization condition-2 were collected at temperatures ranging from 16 to 36 °C, and 19 of the highest resolution data sets across this temperature range were selected for further analysis and are represented in Fig. 6, which plots the B-factor, unit cell volume, and protein volume as a function of temperature. The B-factor displayed an upward trend with an increase in temperature as expected. The break point observed at this temperature in the thermolysin Arrhenius plot [Fig. 1(a)], together with the biphasic break observed between −93 and −73 °C in the plot of B-factor vs temperature in the enzyme ribonuclease A^9^ had led us to predict we would observe a biphasic break of B-factor at 26 °C for thermolysin. However, the 2–2.2 Å resolution limits of thermolysin crystals in our study are an underlying limitation to tightening the distribution of B-factor values at individual temperatures, and therefore may limit our ability to observe a biphasic break in this plot. The plot of unit cell volume vs temperature decreased in a linear fashion across this temperature range [Fig. 6(b)]. The plot of protein volume vs temperature remained constant below and up to the transition temperature of 26 °C. Above this temperature, the protein volume increased [Fig. 6(c)], which is consistent with an increase in conformational mobility of protein side chains. We hypothesize that the increased protein volumes above the break point are suggesting a landscape of protein conformational sampling where thermolysin is more capable of sampling functional conformations. This is consistent with our previous developed htADH model, where the kinetic isotope effect (KIE) is temperature independent above the biphasic transition.^31^

Interestingly, Fraser *et al.*^22^ had observed increases of protein volume when comparing cryo to ambient X-ray crystal structures, which was interpreted in connection with an increase in conformational mobility of side chains. Our results here for thermolysin suggest the biphasic break point at 26 °C is a temperature gateway for increased enzyme mobility.

In contrast to thermolysin crystals from crystallization condition-2, those from crystallization condition-1 did not diffract to high resolution above the biphasic transition temperature. One of the crystals from this condition stopped diffracting at temperatures above the dynamic transition temperature (the no diffraction was described with a resolution of 50 Å) and reversibly regained 2 Å X-ray diffraction after the temperature was re-equilibrated below 26 °C [Fig. 5(c)]. The behavior of this crystallization condition, which diffracted to 2 Å below the biphasic transition temperature, could be caused by a change to crystal lattice contacts, also suggesting a temperature dependent change to protein conformational sampling at the transition temperature. The space group and unit cell parameters were identical between the two crystallization conditions, with the only difference being the variation of the crystallization condition. The content of the solvent channels of the crystals, which are bathed in the makeup of the solution, provides a possible explanation of varied temperature dependent diffraction behavior of these two crystallization conditions.

### Temperature dependent conformational sampling

**FIG. 7.**
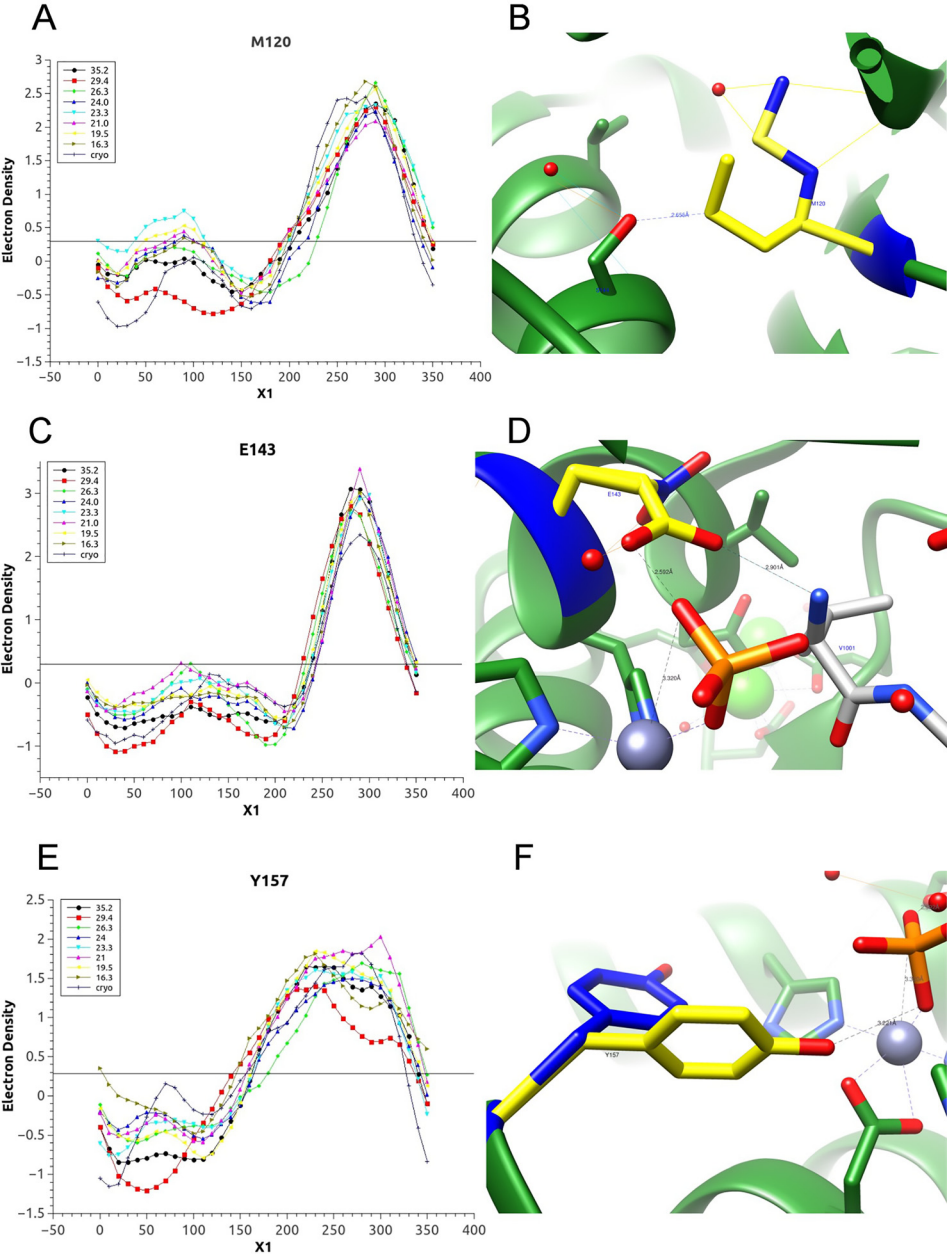
Differences is conformational sampling was displayed below and above the biphasic non-Arrhenius break point for thermolysin. Ringer plots and structures are shown for representative residues from the active site and a remote site. In Ringer plots, the temperatures of 29.4 °C and 35.2 °C, which are above the proposed transition temperature of 26 °C, are shown as red squares and black circles, respectively. (a) The Ringer plot of residue Met120, which is outside the catalytic active site, showed a side chain conformational minority alternate conformation (*X_1_* value of 80°) below the non-Arrhenius break point. (b) The majority conformation of Met120 H-bonded to the Ser144 residue of L114S (modeled) suggests this is the active form since this mutant exhibits a 10-fold increase in catalytic efficiency.^35^ (c) The Ringer plot of Glu143 shows alternate conformations with *X_1_* angles of 100° and 280° at lower temperatures 21.0 °C and 26.3 °C, vs a single *X_1_* angle of 300° at higher temperatures of 29.4 °C and 35.2 °C. (d) The Glu143 rotamer with *X_1_* of 300° (yellow) is the catalytically active rotamer vs the inactive rotamer with a *X_1_* of 100° (blue). (e) The Ringer plot of Tyr157 showed two alternate conformations shown in panel (f) with *X_1_* angles of 210° (yellow) and 310° (blue). The yellow rotamer is the major conformation for structures above the break point. This conformation is directly contributing to the active site, which makes it available to stabilize the transition state of the reaction.

Using the highest resolution X-ray diffraction data sets of thermolysin, the program Ringer was used to create plots of electron density vs side chain torsion angles for residue side chains of interest. Each line represents the electron density distribution over the dihedral angel of the specific residue at one temperature as shown for representative residue Met120, Glu 143 and Tyr157 in Fig. 7.

Several residues in the intermediate flexibility regions, reflected from the previous HDX kinetics, have unique alternate conformations only either above or below the break point. Met120 [Figs. 7(a) and 7(b)], Asn89, and Asn96 have lower occupancy alternate conformation below the break point. Asp261 has a minority alternate conformation only above the break point, and the alternate conformation of the side chain is H-bonded with Lys265 side chain. Asp16, which is remote from the active site, is a residue belonging to one of the loops that displayed increased mobility by HDX experiments,^11^ and it is part of a larger scale hinge bending motion that is needed to bind substrates and release products. Compared to other temperatures analyzed, at 26.3 °C and 29.4 °C the side chain of Asp16 shifted by 50°, which is consistent with the fast HDX kinetics of this residue from previous research.^11^

Glu143 [Figs. 7(c) and 7(d)] is a critical catalytic residue in the proposed thermolysin catalysis mechanism, which activates the water molecule before the hydrolysis reaction.^33,34^ From the Ringer plot of Glu143, we observed an alternate conformation of the Glu143 *X_1_* angle of 100° and 280° at lower temperatures 21.0 °C and 26.3 °C, vs a *X_1_* angle of 300° only at higher temperatures. The alternate rotamer with *X_1_* of 100° was built into the structure with 10% occupancy based on the electron density distribution, followed by structural refinement. The R_free_ dropped from 0.200 to 0.193 after this refinement. At lower temperatures (21.0 °C and 26.3 °C) the non-catalytically active rotamer with low occupancy may contribute to the higher activation energy and higher Arrhenius factor at low temperatures in the Arrhenius plot.

Tyr157 is one of the residues that stabilize the intermediate state of thermolysin by interacting with the substrate peptide carbonyl group. The Ringer plots and structure of Tyr157 side chain showed a 80° rotation at 26.3 °C and 29.4 °C, again indicating a significant change to conformational sampling in this temperature range. In the Ringer plot of Tyr157 [Fig. 7(e)], we were able to observe two alternate conformations of the side chain with different *X_1_* angles, one presumed active and one inactive. The Ringer plots showed the major conformation for structures at higher temperatures of 29.0 °C and 36.0 °C was the active conformation, where the side chain faces the active site, which makes it available to stabilize the transition state of the reaction. Also Tyr157 is in close contact with Asp150, and Asp150Trp has been proven to show a fivefold increase in *k_cat_* in previous published work.^35^ It is possible that the larger size of Trp150 can push Tyr157 from the inactive to active conformation to make it more catalytically favorable. This assumption is consistent with the lower catalytic activation energy at higher temperature from the Arrhenius plot, and the conclusion from a previously published paper which claimed residue-150 plays an important role in the stabilization of the transition state.^36^

Overall, to gain a better understanding of how the structural changes of thermolysin affect the protein function, we used the program Ringer to sample the alternate conformations of the residue side chains of interest over the temperature range of 16–36 °C, and we observed either a more widely distributed electron density or shifting of the dihedral angles in the temperature range of 26.3 °C and 29.4 °C, which indicates an increase in side chain mobility in this transition temperature range. The results further support our hypothesis of a biphasic transition in protein conformational sampling, indicating a biphasic transition exists close to 26 °C in the thermolysin system. The resolution limits of our data sets prohibit our quantitative understanding of the differences observed in a Ringer analysis in terms of discrete rotamer populations, but they lead us to an understanding of qualitative changes. Moving forward, improvement in crystal quality will allow us to collect ambient temperature X-ray diffraction data sets at higher resolution (ideally sub 1.6 Å) and will give a clearer understanding of altered modes of conformational sampling below and above the biphasic break point of 26 °C. The *k_cat_* value we report represents the rate-limiting chemical step of the reaction, which is controlled by enzyme features that activate the attack of the catalytic water molecule.^34^ We conjecture that changes to conformational sampling and dynamics below and above the biphasic break point of thermolysin differentially influences the chemical step of the catalyzed reaction, consistent with the change of the activation energy.

## CONCLUSIONS

The biphasic transition observed at 26 °C for thermolysin provides insight into the relationship of protein structure, dynamics, and function. Our results indicate there is a transition temperature above which thermolysin alters its kinetic function. Our current results strongly suggest a dynamic biphasic transition in protein conformational sampling that has improved catalytic efficiency above this temperature. When the temperature drops below the biphasic transition temperature, thermolysin conformational sampling shifts to a less functionally efficient sampling mode. This could be caused by a network of residues of the enzymes that have a larger population of catalytically efficient alternate conformations above the biphasic break temperature, that could contribute to a larger pool of catalytically competent conformations of the enzyme overall.

In our current study, the resolution of data sets poses a limitation to a full atomic and dynamic understanding of the system. Further studies of ambient temperature crystallography on thermolysin bound with a range of inhibitors and substrate analogs may allow higher resolution structural analysis to be carried out at ambient temperatures. Additionally, our analysis will benefit from new methods at high intensity light sources that allow ambient crystallography to be collected at higher resolution at synchrotron light sources.^37,38^

Furthermore, we predict our observations for thermolysin are general and will apply to other enzymes. Other thermophilic enzymes, in particular, need to be probed for comparable functional and dynamic biphasic break points using kinetics, ambient temperature crystallography, NMR, HDX, and other approaches. A thorough analysis of structural, dynamic, and ultimately computational approaches will provide a better understanding of how networks of residue conformational motions work in a high vs low efficiency sampling mode. The information provided will ultimately lead to a better understanding of the relationship between structure, dynamics, and function, as well as to inform us how to best modulate a protein's function.

## NOMENCLATURE

A: Arrhenius-factor
CD: Circular dichroism
Ea: Activation energy
FAAFA: Furylacryloyl dipeptide N-(3-[2-furyl]acryloyl)-Ala-Phe amide
HDX: Hydrogen/deuterium exchange
ht-ADH: *B. Stearothermophilus* alcohol dehydrogenase
NMR: Nuclear magnetic resonance
